# Supplementary material for: Modeling glioblastoma heterogeneity as a dynamic network of cell states
Source: Mol Syst Biol. 2021 Sep 16;17(9):e10105. doi: 10.15252/msb.202010105 (PMC8444284; doi:10.15252/msb.202010105)
Supplement: Supplementary file 6 — Source Data for Figure 5 [file MSB-17-e10105-s004.zip › Figure5A_sourcedata/GSEA_3071/nosigdb_stateIII.GseaPreranked.1622795155562/NB_KI_CLUSTER9.html]

Details for gene set NB\_KI\_CLUSTER9[GSEA]

|  || Dataset | state33071 |
| Phenotype | NoPhenotypeAvailable |
| Upregulated in class | na\_pos |
| GeneSet | NB\_KI\_CLUSTER9 |
| Enrichment Score (ES) | 0.1742324 |
| Normalized Enrichment Score (NES) | 1.0024897 |
| Nominal p-value | 0.44680852 |
| FDR q-value | 0.43443292 |
| FWER p-Value | 0.999 |
Table: GSEA Results Summary

  

Fig 1: Enrichment plot: NB\_KI\_CLUSTER9      
 Profile of the Running ES Score & Positions of GeneSet Members on the Rank Ordered List

  

| PROBE | GENE SYMBOL | GENE\_TITLE | RANK IN GENE LIST | RANK METRIC SCORE | RUNNING ES | CORE ENRICHMENT || 1 | AL022323.4 |  |  | 509 | -0.255 | -0.0942 | Yes |
| 2 | ZNF169 |  |  | 567 | -0.258 | -0.0723 | Yes |
| 3 | WDR66 |  |  | 584 | -0.259 | -0.0397 | Yes |
| 4 | TMEM201 |  |  | 612 | -0.260 | -0.0098 | Yes |
| 5 | PLAG1 |  |  | 623 | -0.261 | 0.0245 | Yes |
| 6 | AL121832.2 |  |  | 702 | -0.265 | 0.0420 | Yes |
| 7 | CHKB |  |  | 1006 | -0.280 | 0.0041 | Yes |
| 8 | DUS2 |  |  | 1014 | -0.280 | 0.0420 | Yes |
| 9 | TMEM104 |  |  | 1130 | -0.286 | 0.0530 | Yes |
| 10 | SLC9A8 |  |  | 1154 | -0.288 | 0.0879 | Yes |
| 11 | ZNF28 |  |  | 1275 | -0.294 | 0.0987 | Yes |
| 12 | CHRNA5 |  |  | 1284 | -0.294 | 0.1383 | Yes |
| 13 | AHCYL2 |  |  | 1562 | -0.310 | 0.1113 | Yes |
| 14 | PTPN23 |  |  | 1797 | -0.324 | 0.0974 | Yes |
| 15 | PATJ |  |  | 1830 | -0.326 | 0.1353 | Yes |
| 16 | AC026401.3 |  |  | 1985 | -0.334 | 0.1433 | Yes |
| 17 | SIPA1L3 |  |  | 2052 | -0.338 | 0.1742 | Yes |
| 18 | NEMP1 |  |  | 2558 | -0.378 | 0.0986 | No |
| 19 | TRIM66 |  |  | 2871 | -0.405 | 0.0761 | No |
| 20 | KAT14 |  |  | 3014 | -0.422 | 0.0996 | No |
| 21 | SLC35E2A |  |  | 3263 | -0.453 | 0.1002 | No |
| 22 | ZNF606 |  |  | 3456 | -0.494 | 0.1210 | No |
Table: GSEA details [plain text format]

  

Fig 2: NB\_KI\_CLUSTER9: Random ES distribution      
 Gene set null distribution of ES for **NB\_KI\_CLUSTER9**
